# Nuclear Receptors (PPARs, REV-ERBs, RORs) and Clock Gene Rhythms in Goldfish (*Carassius auratus*) Are Differently Regulated in Hypothalamus and Liver

**DOI:** 10.3389/fphys.2022.903799

**Published:** 2022-06-06

**Authors:** Miguel Gómez-Boronat, Nuria De Pedro, Ángel L. Alonso-Gómez, María J. Delgado, Esther Isorna

**Affiliations:** Departamento de Genética, Fisiología y Microbiología, Unidad Docente de Fisiología Animal, Facultad de Biología, Universidad Complutense de Madrid, Madrid, Spain

**Keywords:** circadian system, nr1c, nr1f, *nr1d1*, feeding-fasting cycle, light-dark cycle

## Abstract

The circadian system is formed by a network of oscillators located in central and peripheral tissues that are tightly linked to generate rhythms in vertebrates to adapt the organism to the cyclic environmental changes. The nuclear receptors PPARs, REV-ERBs and RORs are transcription factors controlled by the circadian system that regulate, among others, a large number of genes that control metabolic processes for which they have been proposed as key genes that link metabolism and temporal homeostasis. To date it is unclear whether these nuclear receptors show circadian expression and which *zeitgebers* are important for their synchronization in fish. Therefore, the objective of this study was to investigate whether the two main *zeitgeber*s (light-dark cycle and feeding time) could affect the synchronization of central (hypothalamus) and peripheral (liver) core clocks and nuclear receptors in goldfish. To this aim, three experimental groups were established: fish under a 12 h light-12 h darkness and fed at *Zeitgeber* Time 2; fish with the same photoperiod but randomly fed; and fish under constant darkness and fed at Circadian Time 2. After one month, clock genes and nuclear receptors expression in hypothalamus and liver and circulating glucose were studied. Clock genes displayed daily rhythms in both tissues of goldfish if the light-dark cycle was present, with shifted-acrophases of negative and positive elements, as expected for proper functioning clocks. In darkness-maintained fish hypothalamic clock genes were fully arrhythmic while the hepatic ones were still rhythmic. Among studied nuclear receptors, in the hypothalamus only *nr1d1* was rhythmic and only when the light-dark cycle was present. In the liver all nuclear receptors were rhythmic when both *zeitgeber*s were present, but only *nr1d1* when one of them was removed. Plasma glucose levels showed significant rhythms in fish maintained under random fed regimen or constant darkness, with the highest levels at 1-h postprandially in all groups. Altogether these results support that hypothalamus is mainly a light-entrained-oscillator, while the liver is a food-entrained-oscillator. Moreover, nuclear receptors are revealed as clear outputs of the circadian system acting as key elements in the timekeeping of temporal homeostasis, particularly in the liver.

## Introduction

Nuclear receptors (NRs) belong to a superfamily of ligand-activated transcription factors that regulate gene transcription by modulating essential physiological processes such as growth, development, immunity, and metabolic homeostasis (Kojetin and Burris, 2014; Ribas-Latre and Eckel-Mahan, 2016; Lee et al., 2021). All NRs described up to the present share a great homology in their amino acid sequence with highly conserved domain structures (Solt et al., 2011). Some classical NRs are currently well-known, such as steroid or thyroid receptors. However, there are also a huge number of other less known NRs recently described. Recent studies have advanced in the knowledge of three subfamilies of NRs participating as critical regulators of circadian clock, with significant roles in the regulation of metabolism (Solt et al., 2011; Kojetin and Burris, 2014; Berthier et al., 2021; Lee et al., 2021). These three subfamilies are the nuclear receptor subfamily 1 group C (*nr1c*), commonly named as PPAR; the nuclear receptor subfamily 1 group D (*nr1d*) or REV-ERB, and the nuclear receptor subfamily 1 group F (*nr1f*) or retinoic acid-related orphan receptor (ROR) (Nuclear Receptors Nomenclature Committee, 1999; Weikum et al., 2018).

Most NRs bind to DNA as dimers with another member of the NRs superfamily, and this is the case of PPAR receptors, which form heterodimers with the retinoid X receptors (RXRs). Once the linkage has occurred, the heterodimer PPAR/RXR binds to the PPAR response elements (PPRE) in the promoter of its target genes, inducing their expression (Solt et al., 2011; Chen and Yang, 2014). On their side, REV-ERB and ROR are particular NRs, as they act usually as monomers (Kojetin and Burris, 2014; Cook et al., 2015). REV-ERB competes with ROR for binding to the retinoic orphan receptor binding elements–RORE–, acting REV-ERB as a repressor and ROR as an inductor of genes containing RORE elements (Solt et al., 2011; Chen and Yang, 2014; Ribas-Latre and Eckel-Mahan, 2016; Albrecht and Ripperger, 2018).

As regulators of genes involved in lipid and glucose metabolism (lipid storage and lipogenesis, hepatic fatty acid oxidation, gluconeogenesis, or ketogenesis among several functions), these three subfamilies of proteins provide an essential key for the maintenance of energy homeostasis in metabolic tissues (Chen and Yang, 2014; Pawlak et al., 2015; Vieira et al., 2015; Duszka and Wahli, 2020; Berthier et al., 2021; Lee et al., 2021). Although they were considered orphan receptors for a long time, different endogenous compounds have been emerged as natural ligands for these NRs. Specifically, PPAR ligands are endogenous fatty acids, such as N-acylethanolamines and eicosanoids (Chen and Yang, 2014), being the heme group the main endogenous molecule which reversibly binds to REV-ERBs (Yin et al., 2007; Kojetin and Burris, 2014), while cholesterol is suggested to be a potential reversibly ligand for RORs (Solt et al., 2011). In this sense, these nuclear receptors could act as metabolic sensors.

It is well known that PPARs and REV-ERBs/RORs are the main NRs that link metabolism and circadian system (Vieira et al., 2015; Ribas-Latre and Eckel-Mahan, 2016; Albrecht and Ripperger, 2018). The circadian system is formed by a wide variety of oscillators located in central and peripheral tissues that provides the temporal organization for physiological and behavioral activities enabling the animals to predict environmental cyclic changes. These clocks are closely inter-communicated and generate circadian endogenous rhythms with a period close, but generally not equal, to 24 h (Albrecht, 2012; Schibler et al., 2015; Isorna et al., 2017). The molecular functioning of this set of oscillators is based on transcriptional-translational feedback loops of named clock genes. The main loop is formed by two pairs of clock genes that define two different limbs. The positive limb is represented by the circadian locomotor output cycles kaput (CLOCK) and the brain and muscle ARNT-like 1 (BMAL1) proteins, whose heterodimer acts as an activator transcription factor binding to an E-box rich region in the promoter of the negative limb genes *period* (*per*) and *cryptochrome* (*cry*). Then, the heterodimer of PER:CRY represses the CLOCK:BMAL1 transactivation (Nader et al., 2010; Schibler et al., 2015). The functioning of this molecular mechanisms is well conserved in vertebrates, although several copies of these clock genes have been reported in fishes (Sánchez-Bretaño et al., 2015a; Isorna et al., 2017).

The outputs of the clock occur through the CLOCK:BMAL1 heterodimer which not only promotes the expression of the negative limb genes, but also induces the expression of genes known as clock-controlled genes (CCG), by binding to an E-box rich region in their respective promoters (Albrecht and Ripperger, 2018). These CCGs, which expression follows a 24-h pattern, are considered the outputs of the clocks, and include a wide variety of metabolic, endocrine, and feeding-related genes. Many transcription factors are considered as CCGs, such as the NRs subject of the present study, PPARs, REV-ERBs, and RORs. Besides their role as outputs of endogenous oscillator, these transcription factors also constitute auxiliary loops of the core clock, that stabilize the main loop giving more robustness to the cycling and synchronization of the circadian system (Schibler et al., 2015; Albrecht and Ripperger, 2018), at least in mammals. The possible circadian rhythmicity of these NRs in teleost has not been explored to date.

Although clock circadian oscillations are endogenous, the synchronization of the circadian system to external cues is essential to keeping temporal organization and to predict environmental cyclic changes. The environmental signals that entrain endogenous clocks are named *zeitgeber*s, “time giver” in German (Albrecht, 2012; Tsang et al., 2014; Challet, 2015), being the light-dark (LD) cycle the most important one, that dominantly entrains the central clocks. Clocks synchronized by this *zeitgeber* are named Light-Entrainable Oscillators or LEOs (Mendoza and Challet, 2009; Tahara and Shibata, 2018). The effects of the LD cycle on peripheral organs are not as evident as in the brain. However, feeding time is considered one of the most potent *zeitgeber*, that especially synchronizes the peripheral clocks of the gastrointestinal tract in mammals (Tahara and Shibata, 2018) and in fish (Nisembaum et al., 2012; Isorna et al., 2017; Gómez-Boronat et al., 2018). These peripheral clocks entrained by the feeding-fasting cycles are known as Feeding-Entrainable Oscillators or FEOs (Stephan, 2002; Mendoza and Challet, 2009) and allow tissues to anticipate the food arrival in order to optimize the processes demanded for food digestion, metabolism, and energy storage and utilization (Lamia et al., 2008). In fish, the effects of feeding time on physiological and behavioral aspects are well studied. Feeding time affects daily locomotor activity rhythms (Aranda et al., 2001; Cavallari et al., 2011; Feliciano et al., 2011; Gómez-Boronat et al., 2018); clock genes expression in liver, gut, and brain (López-Olmeda et al., 2009; López-Olmeda et al., 2010; Feliciano et al., 2011; Nisembaum et al., 2012; Tinoco et al., 2014; Gómez-Boronat et al., 2018); and daily profile of circulating cortisol (Montoya et al., 2010; Cowan et al., 2017; Gómez-Boronat et al., 2018; Saiz et al., 2021). However, it is unknown whether feeding time, in addition to entrain clock genes, also drives daily rhythms in PPARs, REV-ERBs, and RORs transcription factors in teleost.

Therefore, the aims of this work were first to study if the NRs rhythms could be of circadian nature in central and peripheral clocks in goldfish (*Carassius auratus*), and second to investigate whether such rhythms are synchronized by the two main *zeitgeber*s (LD cycle and feeding time). To this end, we have studied the effects of an absence of the LD cycle and feeding schedule on 24-h daily patterns of clock genes (*per1a*, *per1b*, *per2a*, *per3*, *clock1a*, and *bmal1a*) and NRs transcripts (*pparα*, *pparγ*, *nr1d1*, *nr1d2 b*, and *rorα a*) in the hypothalamus and the liver in this teleost species. Daily pattern in plasma glucose, as an important metabolic output of the circadian system, was also studied.

## Material and Methods

### Animals and Housing

Goldfish with a body weight (bw) of 13.9 ± 0.4 g were obtained from a local commercial supplier (ICA). Fish were housed in 60 L aquaria with filtered and aerated fresh water (21 ± 2°C), under a 12L:12D photoperiod (lights on at 8 a.m., considered as *Zeitgeber* Time 0, ZT 0). Fish were daily fed at ZT 2 with food pellets (1% bw; Sera Pond Biogranulat). Animals were acclimated during 3 weeks under these conditions before the experiment. All the animal procedures complied with the Guidelines of the European Union Council (UE63/2010) and the Spanish Government (RD53/2013) for the use of animals in research and was approved by the Community of Madrid (PROEX 170.6/20).

### Experimental Design

Fish (*n* = 147, 7/aquaria) were divided into three groups. One group (Group I; *n* = 49) was maintained under a 12L:12D photoperiod and scheduled fed at ZT 2. A second group (Group II; *n* = 49) was maintained under a 12L:12D photoperiod and was randomly fed at fasting-eating intervals of a maximum 24 h. Random feeding times were provided by the random number generator software (RAND function, Microsoft Excel^®^). The third group (Group III; *n* = 49) was maintained at constant dark (24D) and scheduled fed at circadian time 2 (CT 2). One month later, goldfish were sampled every 4 h throughout a 24-h cycle (*n* = 7 fish/sampling time; ZT 3, ZT 7, ZT 11, ZT 15, ZT 19, ZT 23, and ZT 3 on next day—named ZT 3b). Food was offered as scheduled (ZT 2/CT 2) the first day of the experiment, but not the second day before last sampling time (ZT 3b). Thus, the possible effect of food intake on gene expression and circulating glucose levels was tested by comparing fish sampled at the same daily time, but 1-h postprandial (ZT 3) or 25-h fasting (ZT 3b). Blood was collected from the caudal vein of anesthetized animals (tricaine methanesulfonate, MS-222, 0.14 g/L; Sigma-Aldrich), and plasma was obtained after blood centrifugation and stored at −80°C until assay. Fish were then sacrificed by anesthetic overdose (MS-222, 0.28 g/L), and hypothalamus and liver were quickly collected, frozen in liquid nitrogen, and stored at −80°C until analysis.

### Quantification of Plasma Glucose

The circulating glucose was determined using an enzymatic/colorimetric method with commercial kit (GOD-POP; Spinreact), adapted to microplates (Gómez-Boronat et al., 2020).

### Analysis of mRNA Abundance

Total RNA from hypothalamus and liver were isolated using TRI^®^ Reagent (Sigma-Aldrich) and treated with RQ1 RNase-Free DNase (Promega), according to the manufacturer’s instructions. Then, 0.3 μg of total RNA was reverse transcribed into cDNA in a 25 μL reaction volume using random primers (Invitrogen), RNase inhibitor (Promega), and SuperScript II Reverse Transcriptase (Invitrogen). Real-Time quantitative PCRs (RT-qPCRs) were carried out in duplicates in a CFX96™ Real-Time System (Bio-Rad Laboratories, Hercules, USA), using iTaq™ Universal SYBR^®^ Green Supermix (Bio-Rad Laboratories) into a 96-well plate loaded with 1 μL of cDNA and 0.5 μM of each forward and reverse primer in a final volume of 10 μL. Each PCR run also included a 4-points serial dilution curve and non-retrotranscribed-RNA and water as negative control. The RT-qPCR cycling conditions consisted of an initial denaturation at 95°C for 30 s and 40 cycles of a two-step amplification program (95°C for 5 s and 60°C for 30 s). A melting curve was systematically monitored (temperature gradient at 0.5°C/5 s from 70 to 90°C) at the end of each run, to confirm the specificity of the amplification reaction. The Gene Data Bank reference numbers and the primers (Sigma-Aldrich) sequences employed for target genes (clock genes: *per1a*, *per1b*, *per2a*, *per3*, *clock1a*, and *bmal1a*; clock-related NRs: *pparα*, *pparγ*, *nr1d1*, *nr1d2 b*, and *rorα a*) and the reference gene elongation factor 1α (*eef*-*1a1*) are shown in Table 1. The 2^−ΔΔCt^ method (Livak and Schmittgen, 2001) was used to determine the relative mRNA abundance (fold change), normalizing to the group with the lowest level in each gene.

**TABLE 1:** Accession numbers of the genes and primers sequences employed in quantitative RT-qPCR studies.

Gene	Accession Number		Primer Sequence 5’ → 3′	Product (bp)
*per1a*	EF690698	Forward	CAG​TGG​CTC​GAA​TGA​GCA​CCA	155
		Reverse	TGA​AGA​CCT​GCT​GTC​CGT​TGG	
*per1b*	KP663726	Forward	CTC​GCA​GCT​CCA​CAA​ACC​TA	235
		Reverse	TGA​TCG​TGC​AGA​AGG​AGC​CG	
*per2a*	EF690697	Forward	TTT​GTC​AAT​CCC​TGG​AGC​CGC	116
		Reverse	AAG​GAT​TTG​CCC​TCA​GCC​ACG	
*per3*	EF690699	Forward	GGC​TAT​GGC​AGT​CTG​GCT​AGT​AA	130
		Reverse	CAG​CAC​AAA​ACC​GCT​GCA​ATG​TC	
*clock1a*	KJ574204	Forward	CGA​TGG​CAG​CAT​CTC​TTG​TGT	187
		Reverse	TCC​TGG​ATC​TGC​CGC​AGT​TCA​T	
*bmal1a*	KF840401	Forward	AGA​TTC​TGT​TCG​TCT​CGG​AG	161
		Reverse	ATC​GAT​GAG​TCG​TTC​CCG​TG	
*pparα*	AY198322	Forward	CCA​TCC​CGA​CAA​CGA​GTT​CC	121
		Reverse	CAG​CGA​CGT​GTC​TTC​TGT​CT	
*pparγ*	AY894893.1	Forward	TTC​CAC​AGC​TGT​CAG​TCT​CG	201
		Reverse	CAT​GAA​GAT​CTG​TCC​GTA​GG	
*nr1d1*	KU242427	Forward	CGT​TCA​TCT​CAG​GCA​CCA​CT	166
		Reverse	AAC​TGA​CCT​GCA​GAC​ACC​AG	
*nr1d2 b*	MH674345	Forward	AGC​TGC​AAG​CTC​TGA​ACC​TC	164
		Reverse	GTT​GGG​GTG​GTT​CTT​GGT​GA	
*rorα a*	MK033601	Forward	AAG​TCA​TGT​GGC​AGT​TGT​GTG	107
		Reverse	CTG​ATC​ATT​CTG​ACA​GAG​CTC​CA	
*eef-1a1*	AB056104	Forward	CCC​TGG​CCA​CAG​AGA​TTT​CA	101
		Reverse	CAG​CCT​CGA​ACT​CAC​CAA​CA	

per, period; clock1a, circadian locomotor output cycles kaput 1a; bmal1a, brain and muscle ARNT-like 1a; nr1d1, nuclear receptor subfamily 1 group D member 1 (rev-erb α); nr1d2, nuclear receptor subfamily 1 group D member 2 (rev-erb β); ppar, peroxisome proliferator-activated receptor; rorα a, retinoic acid-related orphan receptor alpha paralog a; eef-1a1, elongation factor-1α.

### Data Analysis

A one-way ANOVA followed by the *post hoc* Student-Newman-Keuls (SNK) test was performed to compare gene expression at different sampling points (SigmaPlot 12.0 statistics package). When necessary, data were transformed to logarithmic or square root scale to normalize and to obtain homoscedasticity. A probability level of *p* < 0.05 was considered statistically significant in all tests. Daily (24-h) significant rhythms were determined by Cosinor analysis fitting the data to sinusoidal functions by the least squares method (Duggleby, 1981). The formula used was *f(t)* = *M* + A_*_cos(t_*_π/12-*Φ*), where *f(t)* is the gene expression level at a given time, the mesor (*M*) is the mean value, *A* is the sinusoidal amplitude of oscillation, *t* is time in hours, and *Φ* is the acrophase. Non-linear regression allows the estimation of *M*, *A*, *Φ* and their standard errors (SE), which are calculated on the residual sum of squares in the least-squares fit (Duggleby, 1981; Delgado et al., 1993). Significance of Cosinor analysis was defined by the noise/signal of amplitude calculated from the ratio *SE(A)/A* (Nisembaum et al., 2012). Data were considered to display a significant daily rhythm if fulfil these criteria: *p* < 0.05 by ANOVA, *SE(A)/A <* 0.3 by Cosinor analysis, and *A* > 1 by Cosinor analysis.

## Results

**FIGURE 1:**
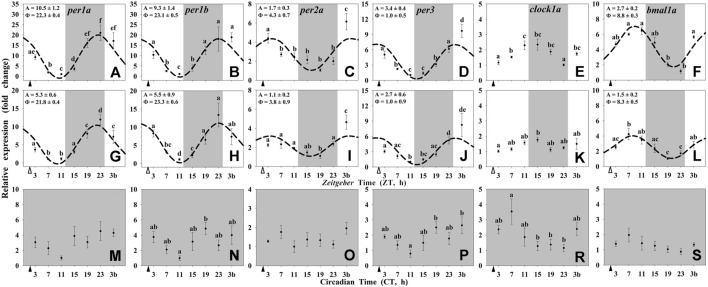
Daily profile of clock genes transcripts in the hypothalamus of goldfish maintained under different lighting conditions and feeding protocols. Grey area indicates the dark period. Feeding time is indicated by triangles in the x-axis (solid, ▲, scheduled feeding at ZT/CT 2; white, ∆, random feeding with the last meal at ZT 2). Data are shown as mean ± SEM (*n* = 5–7) in relative units (2^−ΔΔCt^ method). Different letters indicate significant differences (ANOVA p < 0.05, SNK post hoc test). When Cosinor (*SE(A)/A* < *0.3*) was significant and *A > 1*, periodic sinusoidal functions were represented as dashed curves with the corresponding amplitudes and acrophases (*A* and *Φ*, respectively).

In the hypothalamus of the animals maintained under 12L:12D and scheduled feeding at ZT 2 (Group I) the mRNA abundance of all of the studied clock genes (*per1a*, *per1b*, *per2a*, *per3*, and *bmal1a*) displayed significant 24-h rhythms (Figures 1A–D,F), except *clock1a*, whose transcript showed significant changes through the 24-h (ANOVA) but not a significant rhythm by the Cosinor analysis (Figure 1E). The acrophases of daily rhythms of *per1* genes took place at the end of the dark phase (ZT 22.3 for *per1a* and ZT 23.1 for *per1b*) or at the light onset (ZT 1.0) for *per3*. These rhythmic profiles were in antiphase with that shown by *bmal1a* (ZT 8.8). Hypothalamic *per2a* expression peaked at ZT 4.3 (Figure 1C). The expression profiles of clock genes in the Group II (12L:12D, random feeding) also exhibited daily rhythms in the hypothalamus (Figures 1G–J,L), except for *clock1a* (Figure 1K). The acrophases of all the studied genes were similar to those observed in the Group I, while the amplitudes were slightly reduced. Finally, the hypothalamic rhythmicity of clock genes of the Group III (24D, scheduled feeding at CT 2) was totally lost (Figures 1M–P,R,S).

**FIGURE 2:**
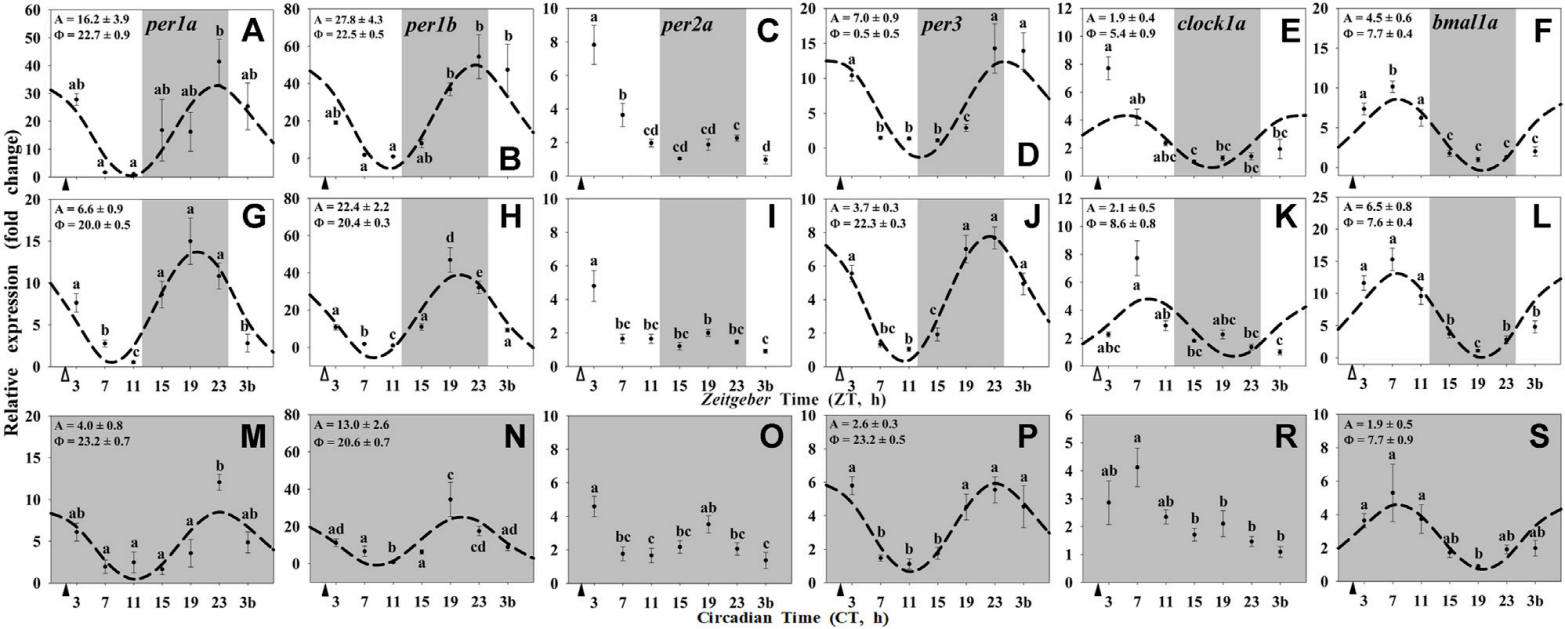
Daily profile of clock genes transcripts in the liver of goldfish maintained under different lighting conditions and feeding protocols. Grey area indicates the dark period. Feeding time is indicated by triangles in the x-axis (solid, ▲, scheduled feeding at ZT/CT 2; white, ∆, random feeding with the last meal at ZT 2). Data are shown as mean ± SEM (*n* = 5–7) in relative units (2^−ΔΔCt^ method). Different letters indicate significant differences (ANOVA p < 0.05, SNK *post hoc* test). When Cosinor (*SE(A)/A < 0.3*) was significant and *A > 1*, periodic sinusoidal functions were represented as dashed curves with the corresponding amplitudes and acrophases (*A* and *Φ*, respectively).

Most of the clock genes in the liver of goldfish (*per1a*, *per1b*, *per3*, *bmal1a*, and *clock1a*) were rhythmically expressed in the three experimental groups (Figure 2), except for *per2a* which did not show daily rhythmicity in any of the studied groups (Figures 2C,I,O), and *clock1a* which lost its 24-h rhythm under constant dark (Group III; Figure 2R). The acrophases of the rhythms of *per* genes in the liver of goldfish reared under 12L:12D and fed at ZT2 (group I) were found at the end of the dark phase (ZT 22.7 for *per1a* and ZT 22.5 for *per1b*; Figures 2A,B) and at the light onset for *per3* (ZT 0.5; Figure 2D), while that for *clock1a* was found at ZT 5.4 (Figure 2E) and at ZT 7.7 for *bmal1a* (Figure 2F). These rhythmic profiles of hepatic clock genes (Figures 2A–F) were similar to those observed in the hypothalamus from the same animals (Figures 1A–F), while the amplitudes were higher than in brain tissue.

In the randomly fed fish (Group II), the acrophases of *per* genes in the liver (Figures 2G–J) were slightly advanced (2 h) compared with the hypothalamus (Figures 1G–J), while the transcripts of *clock1a* peaked 3 h later and the rhythmic profile of *bmal1a* remained unchanged (Figure 2K, L *vs*. Figures 1K,L). Additionally, the amplitudes of clock genes rhythms in randomly fed fishes were reduced (*per* genes; Figures 2G–J) or remained almost unchanged (*clock1a* and *bmal1a* genes; Figures 2K,L) respect to Group I (Figures 2A–F). Regarding the constant dark conditions (Group III), the acrophases of *per* genes were found at the end of the subjective scotophase (CT 21-23; Figures 2M,N,P), and the profile were in antiphase with *bmal1a* (CT 7.7; Figure 2S). Amplitudes of all rhythmic clock genes in the liver of fish under constant darkness (Figures 2M–P,R,S) were strongly reduced compared with animals maintained under a LD cycle (Figures 2A–L).

**FIGURE 3:**
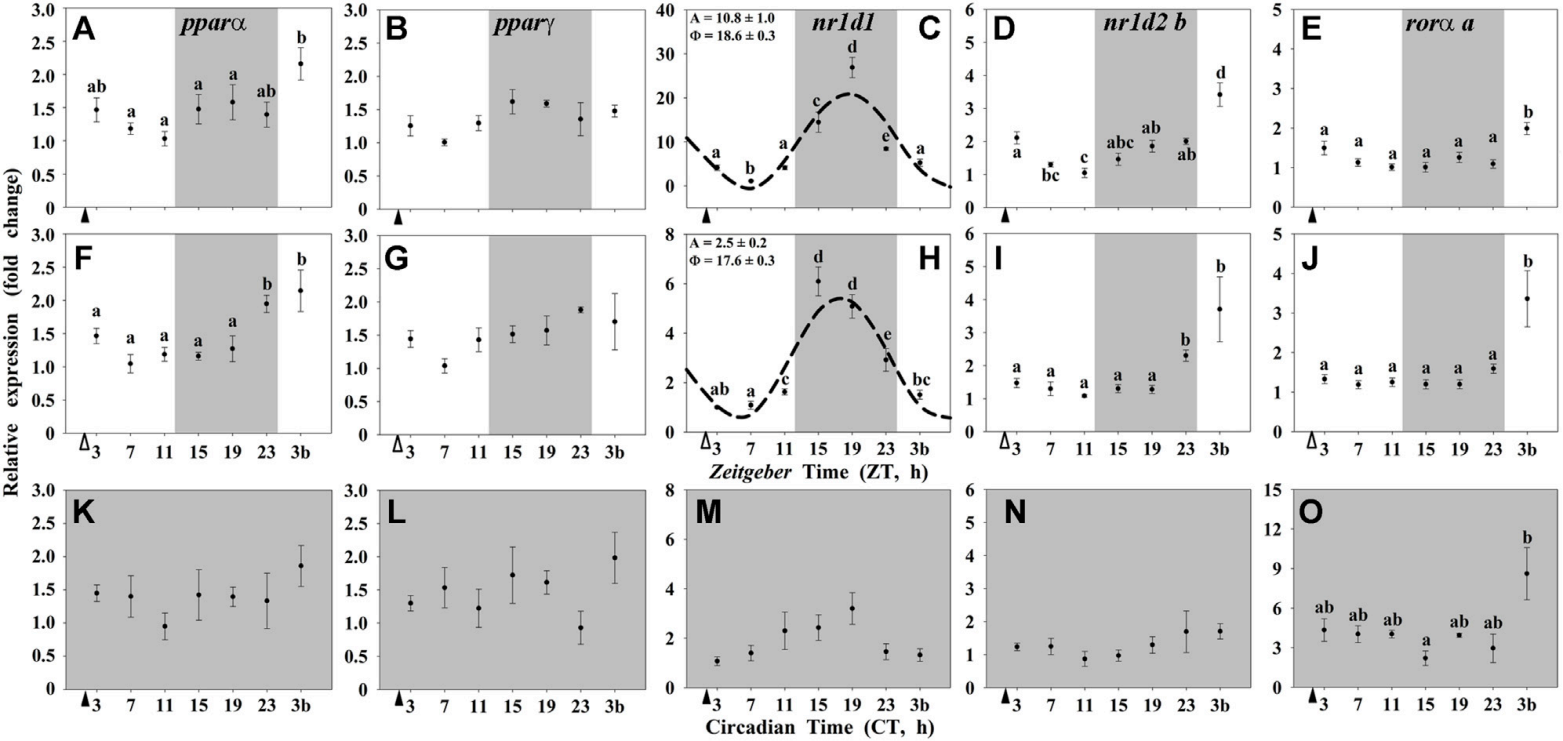
Daily profile of NRs transcripts in the hypothalamus of goldfish maintained under different lighting conditions and feeding protocols. Grey area indicates the dark period. Feeding time is indicated by triangles in the x-axis (solid, ▲, scheduled feeding at ZT/CT 2; white, ∆, random feeding with the last meal at ZT 2). Data are shown as mean ± SEM (*n* = 5–7) in relative units (2^−ΔΔCt^ method). Different letters indicate significant differences (ANOVA p < 0.05, SNK post hoc test). When Cosinor (*SE(A)/A < 0.3*) was significant and *A > 1*, periodic sinusoidal functions were represented as dashed curves with the corresponding amplitudes and acrophases (*A* and *Φ*, respectively).

In the hypothalamus of goldfish, only the NR *nr1d1* showed a significant daily rhythmic expression with a peak at middark (around ZT 17-18; Figures 3). Moreover, such rhythm only occurred when the LD cycle was present (Groups I and II; Figures 3C,H), and showed a high 4-fold reduction in the amplitude in randomly fed animals. Under constant darkness (Group III), any of the NRs studied expressed 24-h rhythmic variations in the hypothalamus of goldfish (Figures 3K–O).

**FIGURE 4:**
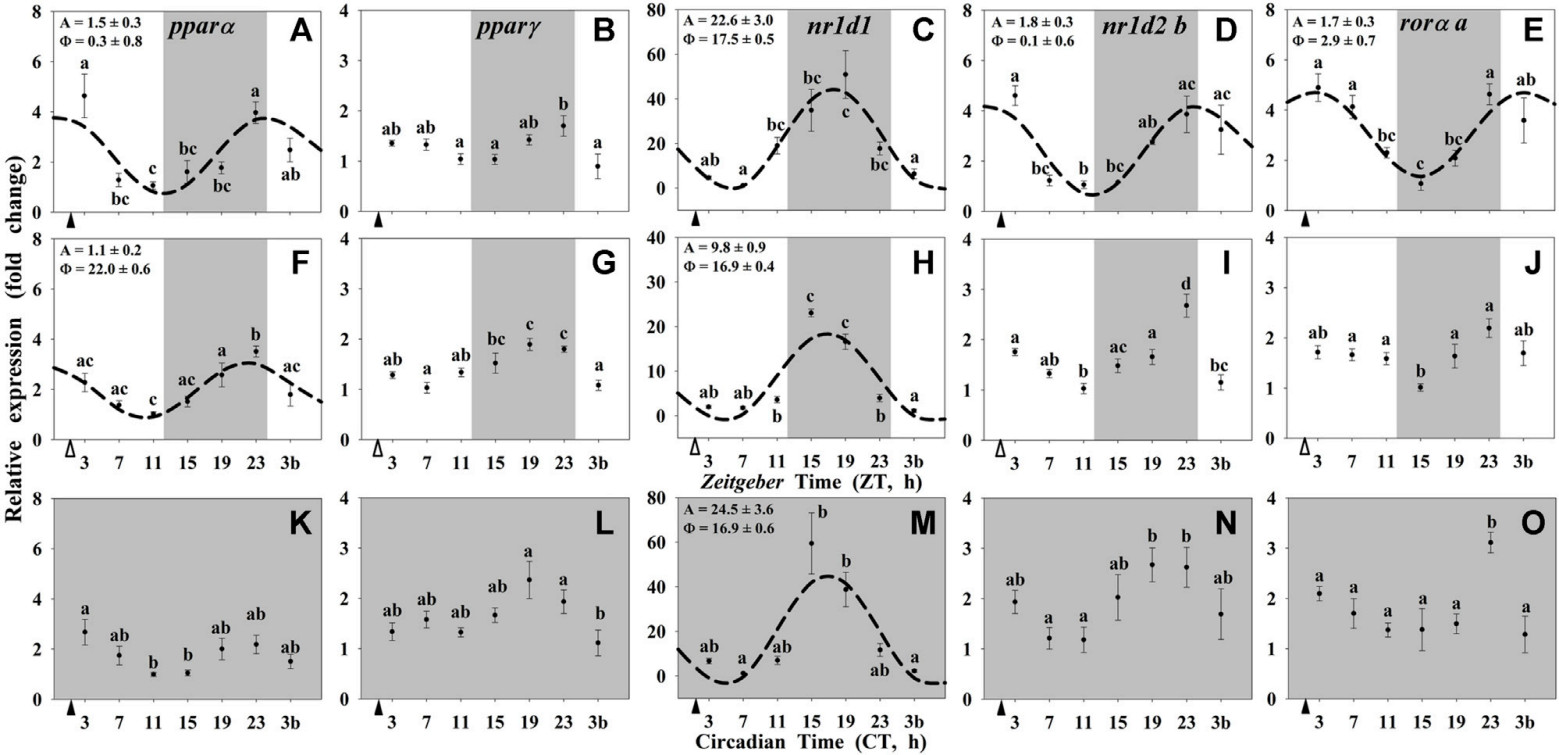
Daily profile of NRs transcripts in the liver of goldfish maintained under different lighting conditions and feeding protocols. Grey area indicates the dark period. Feeding time is indicated by triangles in the x-axis (solid, ▲, scheduled feeding at ZT/CT 2; white, ∆, random feeding with the last meal at ZT 2). Data are shown as mean ± SEM (n = 5–7) in relative units (2^−ΔΔCt^ method). Different letters indicate significant differences (ANOVA p < 0.05, SNK post hoc test). When Cosinor (*SE(A)/A < 0.3*) was significant and *A > 1*, periodic sinusoidal functions were represented as dashed curves with the corresponding amplitudes and acrophases (*A* and *Φ*, respectively).

By contrast, most of the studied NRs in the liver (*pparα*, *nr1d1*, *nr1d2 b*, and *rorα a*) showed daily significant rhythms when both *zeitgeber*s were present (Group I; Figures 4A–E). The acrophases of *pparα* and *nr1d2 b* rhythms occurred at the light onset (ZT 0.3 and ZT 0.1, respectively; Figures 4A,D), *nr1d1* peaked at the middle of the dark period (ZT 17.5; Figure 4C), and *rorα a* displayed its maximum of expression in the early morning (ZT 2.9; Figure 4E). The acrophase of *nr1d1* occurred during the scotophase in the liver (Figure 4C), as in the hypothalamus (Figure 3C), while the amplitude was twice in the peripheral *vs*. the encephalic tissue. These daily patterns of expression remained as significant rhythms only for *pparα* and *nr1d1* in fish randomly fed (Group II, Figures 4F–J), with the acrophases during the dark phase (ZT 22 for *pparα* and ZT 16.9 for *nr1d1*). Comparing with the hypothalamus, the peak of *nr1d1* remained unchanged, but the amplitude was reduced. In Group III (24D, scheduled feeding CT 2), only *nr1d1* was still rhythmic (Figure 4K–O) with similar acrophases and amplitudes as in Group I (Figure 4C).

**FIGURE 5:**
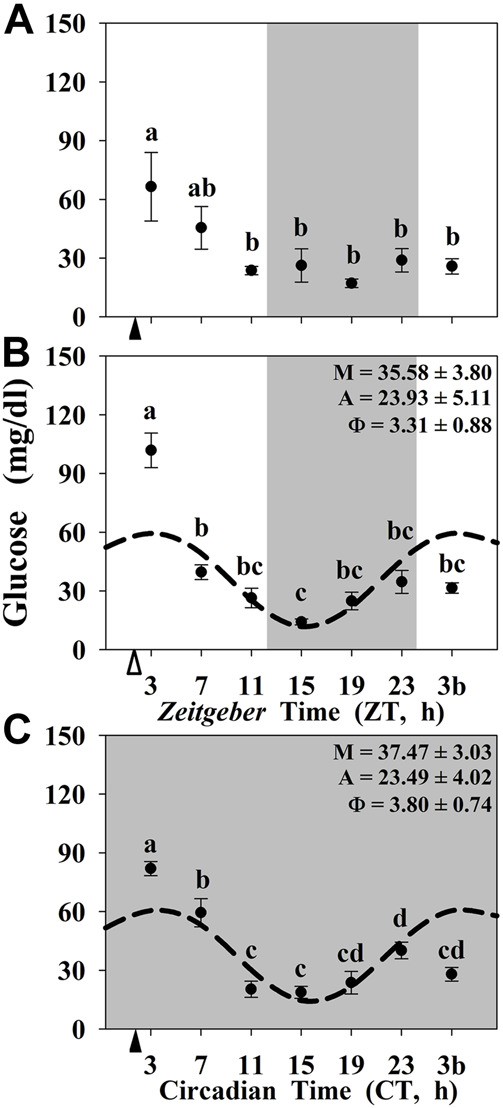
Daily profile of plasma glucose levels in goldfish maintained under different lighting conditions and feeding protocols. Grey area indicates the dark period. Feeding time is indicated by triangles in the x-axis (solid, ▲, scheduled feeding at ZT/CT 2; white, ∆, random feeding with the last meal at ZT 2). Data are shown as mean ± SEM (n = 5–7). Different letters indicate significant differences (ANOVA p < 0.05, SNK post hoc test). When Cosinor (*SE(A)/A < 0.3*) was significant and *A > 1*, periodic sinusoidal functions were represented as dashed curves with the corresponding amplitudes and acrophases (*A* and *Φ*, respectively).

Finally, the circulating levels of glucose are shown in Figure 5. In the three experimental conditions, the highest levels of circulating glucose were observed at 1-h postprandial, while the lowest was found during the dark (resting) period. At the end of the night (or subjective night) an increase in glucose levels, that was extended to the beginning of the photophase (3b sampling point), was observed in all the animals, including unfed fish. However, significant daily rhythms were only found in randomly fed (Figure 5B) and constant darkness maintained animals (Figure 5C).

## Discussion

Results obtained in this work evidence that the absence of one of the main *zeitgeber*s (LD or feeding-fasting cycles) impairs the clock circuitry in a tissue-dependent manner when comparing a central oscillator (hypothalamus) with a peripheral one (liver) in goldfish. Our data also support the idea that the hypothalamus behaves as an authentic LEO, while the liver acts as a true FEO in goldfish (Sánchez-Bretaño et al., 2015a; Isorna et al., 2017; Gómez-Boronat et al., 2018). Nevertheless, the presence of both *zeitgeber*s increases the amplitudes of rhythms in both tissues, probably giving them more robustness to act as timekeepers.

Regarding the core clock of the oscillators, when both *zeitgeber*s are present, both the hypothalamic and the hepatic clocks displayed significant daily rhythms of the negative-limb clock genes (*per*) with acrophases at the end of the scotophase or at the onset of the photophase, while the daily expression rhythms of the positive-limb clock genes (*clock1a* and *bmal1a*) peaked at the end of the photophase. These results show that both the positive and the negative-limb genes are in antiphase, suggesting that these are functional clocks as expected, in accordance with previous results in goldfish also maintained in 12L:12D photoperiod and fed during the photophase (Velarde et al., 2009; Sánchez-Bretaño et al., 2015b; Gómez-Boronat et al., 2018). However, the hypothalamic clock of fish under constant darkness was impaired, as daily rhythms of clock genes expression were lost, making a non-functional oscillator, even when maintaining the scheduled feeding. This is an unexpected result, since a scheduled feeding time under constant light was able to synchronize the daily rhythms of clock genes in central tissues (optic tectum and hypothalamus) of this teleost (goldfish; Feliciano et al., 2011). A possible explanation may lie in the clock gene *per2*. This is a light-inducible gene and a key regulator of the circadian molecular clock in mammals (Kim et al., 2018) and fish (Vatine et al., 2009; Beale et al., 2013; Frøland Steindal et al., 2018). The absence of light is probably the responsible of the very low abundance of *per2* in fish reared under constant darkness, and under these conditions, this gene could not regulate the circadian clock machinery, turning the hypothalamus into a non-rhythmic oscillator. In constant light, a high abundance of *per2* expression is expected (Vatine et al., 2009; Isorna et al., 2017), thus this element would be present, and the clockwork could be not so altered as in constant darkness. On the other hand, the lack of the scheduled feeding did not modify the expression of any hypothalamic clock genes when the LD cycle was present. Together these data are consistent with those observed in mammals (Mendoza and Challet, 2009), and previously reported in fish (López-Olmeda et al., 2010; Gómez-Boronat et al., 2018), and point out that the hypothalamus of goldfish is entrained by the LD cycle behaving as a LEO.

Conversely, the hepatic clock of the fish maintained in darkness seems to remain functional, as daily rhythms of clock genes were preserved. This result agrees with previous results in goldfish under constant light, where a scheduled feeding synchronizes daily rhythms of clock genes in liver and intestine (Feliciano et al., 2011; Nisembaum et al., 2012). However, when the LD cycle was present, such rhythms exhibited greater amplitudes, supporting that both external *zeitgebers* (LD and feeding-fasting cycles) work together sustaining the molecular clock mechanism, at least in the gastrointestinal tissues (Nisembaum et al., 2012; Sánchez-Bretaño et al., 2015a). All current data support the idea that the liver is entrained by the feeding-fasting cycle, acting as a FEO in both, mammals and fish (Mendoza and Challet, 2009; López-Olmeda et al., 2010; Gómez-Boronat et al., 2018). In fact a single temporal change in the food availability can resynchronize peripheral clocks, such as the liver in fish (Feliciano et al., 2011; Gómez-Boronat et al., 2018) as well as in mammals (Lamia et al., 2008; Patton and Mistlberger, 2013).

It is worth noting that food-derived molecules, lipid-soluble nutrients, and hormones can activate transcription factors of the nuclear receptor family, such as REV-ERBs, RORs, and PPARs, among many others (Duez and Staels, 2009; Albrecht and Ripperger, 2018). Therefore, it was expected that these nuclear receptors, that are key elements of the biological clocks’ machinery, were also driven by feeding time. This seems to be the case in the liver, but not in the hypothalamus of goldfish, where the studied nuclear receptors, as clock-controlled genes, seem to be mainly driven by the clock itself, instead of by putative metabolic ligands. Our results showed that in the hypothalamus, only *nr1d1* (*rev-erb*α) displayed significant daily rhythms under a LD cycle in both feeding conditions, scheduled and random feeding. Recently a rhythm in *nr1d1* has been reported in the whole brain in Atlantic salmon (*Salmo salar*) reared under a 12L:12D photoperiod with acrophases during the middle-late scotophase (Bolton et al., 2021) as in goldfish. Interestingly, when goldfish were maintained under constant darkness, such rhythm disappeared, and any nuclear receptor mRNA had oscillations, which coincides with the loss of the clock gene rhythms in these animals and reinforces the hypothesis that the hypothalamus is a classical LEO. The existence of *nr1d1* rhythms driven by the LD cycle in the hypothalamus, but not entrained by feeding-fasting cycle, suggest that other functions beyond the known metabolic role of REV-ERBα are probably linked to this transcription factor in the hypothalamus. Among such putative functions, it has been proposed that REV-ERBα acts as a repressor of the key enzyme for dopamine synthesis, tyrosine hydroxylase, in mammals (Chung et al., 2014; Mendoza, 2019). This action could be a link for a crosstalk between the circadian system and brain functioning by modulating anxiety, addiction and the hedonic system, among others (Banerjee et al., 2014; Chung et al., 2014; Mendoza, 2019). Putative targets of REV-ERBα in fish brain and its relevance for the temporal control of neural functions need to be explored in the future.

On the other hand, all the nuclear receptors showed daily rhythmic patterns of expression in the liver of goldfish when both *zeitgeber*s were present, except *pparγ*. Interestingly, in random-fed fish only *pparα* and *nr1d1* were rhythmic and the latter in the 24D fish. The scheduled feeding in both conditions, 12L:12D and 24D, preserved high amplitudes of expression of *nr1d1*, while in random-fed fish the amplitude was considerably diminished. These results also support the idea that the liver of goldfish woks as a FEO mainly driven by feeding time (Isorna et al., 2017; Gómez-Boronat et al., 2018), and that REV-ERBα is an important element to maintain the temporal homeostasis in this organ.

In the last decades, it is increasingly being developed and established the idea that the circadian system not only controls the metabolic regulation as an output, but also the metabolic status can signals back to the clocks in order to reinforce circadian rhythmicity (Reinke and Asher, 2016; Kolbe et al., 2018; Reinke and Asher, 2019). Indeed, at least 20% of the whole transcriptome in peripheral organs of mouse are rhythmic (Zhang et al., 2014; Panda, 2016), including genes involved in lipid and glucose metabolism, and most of the transcription factors belonging to the nuclear receptor family (Yang et al., 2006). In fish, around 15% of 13,939 genes studied in sea bream also were also rhythmic, including genes of the molecular clock, metabolic genes, and nuclear receptors (Yúfera et al., 2017).

The daily rhythms shown by nuclear receptors in the liver of goldfish, and particularly, the rhythmic expression of *nr1d1* even in constant darkness, suggest their possible implication in the regulation of the temporal homeostasis. Complementary to their contribution to the circadian system, all the described nuclear receptors exert multiple regulatory functions on several metabolic pathways, being the most important REV-ERBα and PPARα (Yang et al., 2007; Kojetin and Burris, 2014; Pawlak et al., 2015; Berthier et al., 2018). These nuclear receptors could play a key role in the control of hepatic glucose and lipid metabolism, which is supported by the preserved rhythmic expression of REV-ERBα in the liver, but not in the hypothalamus of goldfish under constant darkness. In this sense, daily rhythm of *nr1d1* expression in the liver of goldfish peaked at the mid-scotophase (or subjective), i.e., during the rest phase, as these animals were diurnal (personal observations). In addition, the lowest levels of plasma glucose occurred during the scotophase, matching with this maximum hepatic expression of *nr1d1*, which might be explained by the effects that this nuclear receptor exerts on glucose metabolism during fasting/resting phase observed in mammals, downregulating the expression of the gluconeogenic enzymes (PEPCK and G6Pase) and modulating the actions of insulin in mammals (Yin et al., 2007; Vieira et al., 2015; Berthier et al., 2018) and fish (Saiz et al., 2022). On the other hand, plasma glucose raised at the end of the night peaking few hours after the mealtime. Such increment before light onset coincides with the daily rhythm of *rorα* in the liver of goldfish in presence of both *zeitgebers.* In *rorα* ^−/-^ mice a reduced blood glucose levels has been proposed (Kang et al., 2007). It could be possible that hepatic ROR*α* increases plasmatic glucose also in goldfish.

The other nuclear receptor largely involved in the hepatic metabolism is the *pparα*, which showed a daily rhythm of expression with the acrophase placed only a few hours before the last meal in fish exposed to a light/dark cycle, with either scheduled or random feeding. These results are in agreement with those observed in mammals, and support the possible role of this nuclear receptor as responsible for preparing the liver to be able to respond to the lipids that income with the food (Berger and Moller, 2002; Chakravarthy et al., 2009; Liu et al., 2014; Pawlak et al., 2015). Moreover, PPARα is upregulated during the rest phase of the animals, peaking at the end of daytime in nocturnal mammals (Yang et al., 2006; Chen et al., 2010), or at the end of nighttime in diurnal fish (Paredes et al., 2014). During this resting and fasting states, animals obtain energy from increasing the hepatic fatty oxidation (Ribas-Latre and Eckel-Mahan, 2016), metabolic process that is promoted by PPARα in mammals (Chen and Yang, 2014; Liu et al., 2014). Current PPARα expression rhythms confirm our previous results (Gómez-Boronat et al., 2019) and are in agreement to those observed in mammals, suggesting preserved functions throughout the phylogeny.

Interestingly, we observed that the postprandial peak of circulating glucose is overlapped with the basal daily rhythms of this metabolite, which can be observed only when one *zeitgeber* (LD cycle or scheduled feeding) was present. In the three experimental conditions, we detect significant differences between 1-h postprandial (ZT 3/CT 3) and 25-h fasting (ZT 3b/CT 3b) regardless of the photoperiod and scheduled feeding conditions. These data suggest that daily glucose rhythms are directly controlled by the feeding in fish, as seen in mammals (Kasanen et al., 2018). However, this is not the case for NRs expression. In contrast to circulating glucose, we did not obtain any difference between 1-h postprandial and 25-h-fasting in the gene expression of NRs either in the liver or in the hypothalamus of goldfish. Indeed, in the 24D fish neither the hypothalamic core clock genes nor the studied NRs show daily oscillations. However, the hepatic clock genes and *nr1d1* were rhythmic in these animals reared in 24D as well as in the absence of a scheduled feeding. All these obtained data point to NRs being clear outputs of the circadian system, being influenced by the core clock of the oscillators, and not being masked by the feeding-fasting cycle.

In summary, our results evidence the circadian nature of the hepatic oscillator as well as some nuclear clock-related receptors in teleost fish and reinforce the idea that the liver is mainly synchronized by the feeding-fasting cycle, while the light-dark cycle is the main *zeitgeber* entraining the clock genes in the hypothalamus of goldfish. In addition, and bearing in mind the many implications they have, nuclear receptors are being discovered as real outputs of the circadian system acting as very important molecules for the timekeeping of temporal homeostasis, and independently of the energy status in fish.

## Data Availability

The datasets presented in this study can be found in online repositories. The names of the repository/repositories and accession number(s) can be found below: https://www.ncbi.nlm.nih.gov/genbank/, MK033601 https://www.ncbi.nlm.nih.gov/genbank/, MH674345.
